# Acute hepatitis in a patient with multiple biliary hamartomas

**DOI:** 10.1093/omcr/omad072

**Published:** 2023-09-25

**Authors:** Trung V Hoang, Hoang Anh T Van, Huan T Hoang, Hoai T Vo, V Chansomphou

**Affiliations:** Department of Radiology, Thien Hanh Hospital, Buon Ma Thuot, Vietnam; Department of Radiology, Thien Hanh Hospital, Buon Ma Thuot, Vietnam; Department of Radiology, Thien Hanh Hospital, Buon Ma Thuot, Vietnam; Department of Radiology, Tam Tri Nha Trang General Hospital, Nha Trang, Vietnam; Department of Radiology, Savannakhet Medical-Diagnostic Center, Kaysone Phomvihane, Laos

##  

A 53-year-old woman with no medical history was hospitalized with right hypochondriac pain for 3 days. Liver function tests included aspartate aminotransferase of 102 IU/L (normal, 0–40), alanine aminotransferase of 114 IU/L (normal, 0–40) and gamma-glutamyl transpeptidase 145 IU/L (normal, 12 to 64). Hepatitis virus tests were negative. She denied any history of previous hepatitis and history of recent drug. Ultrasonography showed multiple small hypoechoic lesions 2–10 mm in diameter with multiple cometary tail echoes. Computed tomography (CT) scan of her abdomen shortly thereafter revealed numerous small-sized, low-density lesions in the entire liver parenchyma and no enhancement was observed in these lesions (Fig. 1). Because there were no suspicious lesions on ultrasound and CT images, the patient was not subjected to further magnetic resonance imaging. The patient was prescribed the usual liver tonics and maintained for 2 months. Liver function values gradually decreased and returned to normal after 6 weeks of taking the drug. The patient was followed up for 2 years after that; during the follow-up, the laboratory parameters were within the normal limits and the hepatobiliary ultrasound image showed multiple biliary hamartomas that did not change over time. After combining clinical symptoms, laboratory findings and imaging studies, the end result of this case was acute hepatitis and the incidental finding of multiple biliary hamartomas.

**Figure 1:**
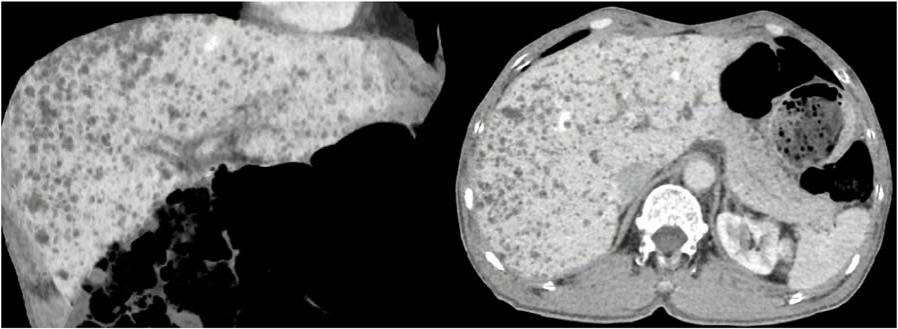
Coronal and axial contrast-enhanced CT images in the late arterial phase show multiple small low-attenuation lesions without enhancement scattered in the whole liver.

Multiple biliary hamartomas (Von Meyenburg complexes) are benign congenital bile duct malformations. These lesions are supposed to arise from failed involuted embryonic bile ducts. Histologically, they are cystic lesions consisting of focal collections of duct structures lined by a single layer of columnar or cuboidal epithelium and submerged in abundant fibrous stroma. In most cases, they are asymptomatic and detected incidentally at imaging, laparotomy or autopsy. Multiple biliary hamartomas have very low incidence ranging from 0.69 to 5.6% in the autopsy series and 0.6% in the needle biopsy seriesation. Although they are usually asymptomatic, a few cases have been reported in association with malignant transformation. They are easily diagnosed on ultrasound when a comet tail is present [1, 2].

On CT, multiple biliary hamartomas appear as multiple hypoattenuating structures less than 10 mm in size and without contrast enhancement. Their differential diagnoses include (i) liver metastasis (appear with different sizes from small to large, unevenly distributed, various degrees of enhancement, no sign of comet tail on ultrasound); (ii) diffuse primary hepatocellular carcinoma (usually occurs in patients with chronic liver disease or cirrhosis, not shown as cystic lesions on ultrasound, no sign of comet tail on ultrasound, various degrees of enhancement); (iii) simple hepatic cysts (round shape, random distribution, larger in diameter and fewer in number, no sign of comet tail on ultrasound); (iv) polycystic liver disease (round shape, random distribution, various sizes, no sign of comet tail on ultrasound, often associated with polycystic kidney); (v) Caroli disease (saccular dilatations of intrahepatic duct, dysmorphic liver with gross dilatations on CT, central dot sign); and (vi) hepatic abscesses (infection situation, poorly demarcated with variable appearance, central necrosis, peripherally enhancing, sometimes contain gas) [1–3].

## Data Availability

All data generated or analyzed during this study are included in this article.
